# Optimization of medium compositions to improve a novel glycoprotein production by *Streptomyces kanasenisi* ZX01

**DOI:** 10.1186/s13568-016-0316-7

**Published:** 2017-01-03

**Authors:** Yong Zhou, Yu-Bo Sun, Hong-Wei He, Jun-Tao Feng, Xing Zhang, Li-Rong Han

**Affiliations:** 1 Research and Development Center of Biorational Pesticides, Northwest A & F University, Yangling, 712100 Shaanxi China; 2 Shannxi Research Center of Biopesticides Engineering and Technology, Northwest A & F University, Yangling, 712100 Shannxi China

**Keywords:** *Streptomyces*, Glycoprotein, Response surface methodology, Anti-TMV, Optimization

## Abstract

*Streptomyces kanasenisi* ZX01 was found to produce a novel glycoprotein GP-1 previously, which was secreted into medium and had significant activity against tobacco mosaic virus. However, the low production of GP-1 by strain ZX01 limited its further studies. In order to improve the yield of GP-1, a series of statistical experimental design methods were applied to optimize medium of strain ZX01 in this work. Millet medium was chosen to be the optimal original medium for optimization. Soluble starch and yeast extract were identified as the optimal carbon and nitrogen source using one-factor-at-a-time method. Response surface methodology was used to optimize medium compositions (soluble starch, yeast extract and inorganic salts). A higher yield of GP-1 was 601.33 µg/L after optimization. The optimal compositions of medium were: soluble starch 13.61 g/L, yeast extract 4.19 g/L, NaCl 3.54 g/L, CaCO_3_ 0.28 g/L, millet, 10 g/L. The yield of GP-1 in a 5 L fermentor using optimized medium was 2.54 mg/L, which is much higher than the result of shake flask. This work will be helpful for the improvement of GP-1 production on a large scale and lay a foundation for developing it to be a novel anti-plant virus agent.

## Introduction

*Streptomyces* is a famous prokaryotic gram-positive mycelial soil bacteria and has become one of the most important microbial resources these years, owing to the ability to produce many kinds of natural products, especially antibiotics (Demain and Sanchez 2009; Watve et al. 2001). Some antibiotics showed great activity against plant virus, such as tunicamycin by *Streptomyces lysosuperificus* (Takatsuki et al. 1971), herbimycin B by *Streptomyces hygroscopicus* (Iwai et al. 1980), ningnanmycin by *Streptomyces noursei* var*. xichangenisi* (Deng et al. 2004), cytosinpeptidemycin by *Streptomyces achygroscopicus* var. *liaoningensis* (Zhu et al. 2005). Non-antibiotics like polysaccharide (He et al. 2010), protein (Gomes et al. 2001) and glycoprotein (Nwodo et al. 2012) also can be produced by *Streptomyces*. However, it was rarely reported that these biopolymers produced by *Streptomyces* had high activity against plant virus.

*Streptomyces kanasenisi* ZX01 (CGMCC 4893) was isolated from soil around Kanas Lake, Xinjiang Province, China. Our previous research indicated that strain ZX01 can produce a novel glycoprotein (GP-1) with significant activity against some plant virus, especially tobacco mosaic virus (TMV) (Han et al. 2015). GP-1 was a heat-sensitive glycoprotein with approximate 8.5 kDa molecular weight, which contained 40.23% carbohydrate with N-linked and O-linked glycan (Zhang et al. 2015). Due to extremely low production of GP-1 and long fermentation time of strain ZX01, this is necessary to improve GP-1 yield in batch culture.

Nutrition plays a significant role in the process of microorganism producing secondary metabolites, not only because limiting the supply of some essential nutrients is an effective way to restrict growth but also because the choice of limiting nutrient can have specific metabolic and regulatory effects. To achieve the maximum yield, it is necessary to design an appropriate fermentation medium in an efficient fermentation process. There is usually a relationship between the medium compositions and secondary metabolites (Azma et al. 2011; Elibol 2004).

Different statistical design methods can be used to optimize the fermentation medium. The conventional method of one-factor-at-a-time optimization need to keep the other factors constant and changes one independent variable. This method is not only time consuming, but also can’t describe the interactions between different factors, leading to unreliable results. These limitations of one-factor-at-a-time method can be replaced by response surface methodology (RSM) (Sayyad et al. 2007; Zhao et al. 2013). Optimization through RSM is a common practice in biotechnology. Various research workers have applied this technique, especially for the optimization of culture conditions, the determination of optimal values for processing parameters such as pH, temperature and aeration (Kalil et al. 2000). RSM, which includes factorial design and regression analysis, can be used to build models to determine relationship, select the optimal conditions of the variables for a desirable response, and estimate the interactions between a set of controlled experimental factors (Muntari et al. 2012).

The objective of the present research was to optimize the fermentation medium of strain ZX01 for the maximum yield of GP-1 using both one-factor-at-a-time optimization and response surface methodology. The conventional one-factor-at-a-time method was applied to screen the medium compositions, such as carbon sources and nitrogen sources. Optimal value of these compositions was obtained by response surface methodology. Moreover, a further study on scale-up fermentation was carried out in 5 L bench fermentor to explore primarily the possibility of scale-up of GP-1 production from shake flask to fermentor.

## Materials and methods

### Strain

*Streptomyces kanasenisi* ZX01 obtained from Research and Development Center of Biorational Pesticide, Yangling, China, was isolated from soil of Kanas Lake, Xinjiang Province, China. Strain ZX01 is registered at China General Microbiological Culture Collection Center (CGMCC) under strain number CGMCC 4893. The strain was maintained on Gause’s No.1 agar medium and subcultured at a month interval, or was stored in 20% glycerol at −70 °C.

### Inoculum preparation

Inoculum was prepared by inoculating a loopful of strain ZX01 growing on Gause’s No. 1 agar plate for 72 h into a 250 mL flask containing 100 mL Gause’s No. 1 liquid medium. The compositions of Gause’s No. 1 liquid medium were (g/L): soluble starch, 20; NaCl, 0.5; FeSO_4_·7H_2_O, 0.01; K_2_HPO_4_, 0.5; KNO_3_, 1; MgSO_4_·7H_2_O, 0.5. Medium was adjusted to a final pH of 7.0 to provide suitable conditions for growth of strain ZX01. The flasks were incubated at 28 °C on a shaker at 180 rpm for 72 h. The inoculum quantity was controlled at 5% in all the fermentation experiments.

### Extraction and measurement of GP-1

The fermentation broth was centrifuged at 10,000 rpm for 20 min to separate the precipitate and supernatant. The supernatant was concentrated to a volume of 10 mL by rotary evaporator and then precipitated by adding 4-fold volumes of ethanol at 4 °C. The precipitate was redissolved in distilled water (10 mL) and centrifuged (10,000 rpm, 10 min) again to remove those water-insoluble materials. The supernatant was subjected to DEAE-52 Cellulose anion-exchange column (2 cm × 60 cm) eluted with deionized water first, and then with 0.1 M NaCl at a flow rate of 5 mL/min. The 0.1 M NaCl fraction was collected and centrifuged (10,000 rpm, 15 min) with centrifugal filter devices (3K, 0.5 mL) to remove NaCl. The fraction was subjected to HiTrap™ Con A 4B eluted with binding buffer (20 mM Tris–HCl, 0.5 M NaCl, 1 mM MnCl_2_, 1 mM CaCl_2_, pH 7.4) and elution buffer (0.1 M methyl-α-d-glucoside, 20 mM Tris–HCl, 0.5 M NaCl, pH 7.4) sequentially at a flow rate of 1 mL/min. The fraction eluted with elution buffer that contained GP-1 was concentrated to 100 µL and evaluated by high performance liquid chromatography (HPLC).

The concentration of GP-1 was analyzed by a HPLC (Waters, USA) with a gel filtration column (TSK-gel G2000SWXL, 7.8 × 300 mm, 5 µm, TOSOH, Japan) and a 996 photodiode array detector at 280 nm. HPLC was performed on 10 µL sample with 20% acetonitrile at a flow rate of 0.5 mL/min and 28 °C. GP-1 purified previously (purity >99%) was diluted to 10, 5, 2.5, 1.25, 0.625 and 0.3125 mg/mL as standards.

### Anti-TMV activity assay

The anti-TMV activity was tested by half-leaf method. The fermentation broth diluted to one twentieth was equally mixed with TMV (50 µg/mL). After 10 min, the mixture was mechanically inoculated onto the left side of the leaves of *Nicotiana glutinosa* as the treatment, while the right side of the leaves was inoculated with a mixture of distilled water and TMV as the negative control. *N. glutinosa* were kept in a culture chamber at 28 °C for 2–3 days, and then the number of local lesions on the leaves was recorded. The TMV inhibition rate was calculated as follows:

1 $${\text{Inhibition rate}}\left( \% \right) = \left( {1 - \frac{T}{C}} \right) \times 100\%$$

where *T* is the average number of local lesions of treatment, *C* is the average number of local lesions of negative control. All experiments were conducted in triplicate. TMV was stored in systemic host *Nicotiana tabacum* K_326_ and purified as described by Gooding and Hebert (1967).

### Selection of the optimal fermentation medium

10 different media were used to find the optimal fermentation medium. The compositions of medium (g/L): bean broth medium (soybean leach liquor, 20; soluble starch, 20; yeast extract, 5; peptone, 2; NaCl, 5; CaCO_3_, 2); modified bean broth medium (soybean leach liquor, 20; soluble starch, 5; sucrose, 10; yeast extract, 2; peptone, 2; NaCl, 2; K_2_HPO_4_, 0.5; MgSO_4_∙7H_2_O, 0.5; CaCO_3_, 3); millet medium (millet leach liquor, 10; glucose, 10; peptone, 3; NaCl, 2.5; CaCO_3_, 0.2); Gause’s No.1 medium (soluble starch, 20; NaCl, 0.5; FeSO_4_∙7H_2_O, 0.01, K_2_HPO_4_, 0.5; KNO_3_, 1; MgSO_4_∙7H_2_O, 0.5); ISP1 (sucrose, 30; NaNO_3_, 2; K_2_HPO_4_, 1; MgSO_4_∙7H_2_O, 0.5; KCl, 0.5; FeSO_4_∙7H_2_O, 0.01); ISP2 (yeast extract, 4; malt extract, 1; glucose, 4; trace salt solution, 1); ISP3 (oatmeal leach liquor, 20; trace salt solution, 1); ISP4 (soluble starch, 10; NaCl, 1; KH_2_PO_4_, 1; MgSO_4_∙7H_2_O, 1; (NH_4_)_2_SO_4_, 2; CaCO_3_, 2; trace salt solution, 1); ISP5 (l-Asparagine, 1; glycerol, 10; KH_2_PO_4_, 1; trace salt solution, 1); PDA (potato leach liquor, 200; glucose, 20). The media were mixed fully and then sterilized at 120 °C for 30 min. 5 mL inoculum was inoculated into 100 mL medium in 250 mL flask. All the flasks were incubated at 28 °C on a shaker at 200 rpm for 7 days. All experiments were conducted in triplicate.

### Selection of the optimal carbon and nitrogen source

One-factor-at-a-time method was used to investigate the best carbon and nitrogen source. We used 7 different carbon sources and 7 different nitrogen sources (Table 3) to replace corresponding carbon and nitrogen source in the optimal fermentation medium while other compositions were kept constant at their original concentration. All experiments were conducted in triplicate.

### Experiments design by response surface methodology

Response surface methodology (RSM) based on central composite design (CCD) was used to optimize optimal levels of medium compositions. The concentrations of carbon source (*X*_1_), nitrogen source (*X*_2_) and inorganic salts (*X*_3_) were selected as the independent variables (main factors). Inorganic salts were defined as total concentration of inorganic salts of medium and kept original proportion. The levels of the variables are presented in Table 1. The total number of experimental combinations was estimated according to the equation:

2 $$N = 2^{k} + 2k + n_{0}$$

where *N*, *k* and *n*_0_ are the number of experimental combinations, the number of the variables and the number of repetitions of experiments at the central point, respectively.

**Table 1:** The levels of the independent variables through CCD

Factors	Variables	Levels				
		−1.682	−1	0	1	1.682
Soluble starch (g/L)	*X*_1_	5.0	7.0	10.0	13.0	15.0
Yeast extract (g/L)	*X*_2_	1.3	2.0	3.0	4.0	4.7
Inorganic salts (g/L)	*X*_3_	1.0	1.7	2.7	3.7	4.4

*x*_i_ = coded value of the variable *X*_i_

*x*_1_ = (soluble starch-10)/3; *x*_2_ = (yeast extract-3)/1; *x*_3_ = (inorganic salts-2.7)/1

A total of 20 experiments were performed, including 2^3^ cube points, 6 axial points and 6 repetitions. The selected independent variables (*X*_i_) were coded as *x*_i_ according to the equation:

3 $$x_{\text{i}} = \frac{{X_{\text{i}} - \bar{X}_{\text{i}} }}{{\Delta X_{\text{i}} }}\left( {{\text{i}} = 1, 2, 3, \ldots {\text{k}}} \right)$$

where *x*_i_ is the coded value of the variable, *X*_i_ is the actual value of the variable, $\bar{X}_{\text{i}}$ is the actual value of the variable at the central point and $\Delta X_{\text{i}}$ is step change value.

The mathematical relationship between the response variable (the yield of GP-1) and the independent variables can be described by the following equation:

4 $$Y = b_{0} + \mathop \sum \limits_{\text{i}} b_{\text{i}} x_{\text{i}} + \mathop \sum \limits_{\text{i}} \mathop \sum \limits_{\text{j}} b_{\text{ij}} x_{\text{i}} x_{\text{j}} + \mathop \sum \limits_{\text{ii}} b_{\text{ii}} x_{\text{i}}^{2}$$

where *Y* is the predicted response, *b*_0_, *b*_i_, *b*_ij_ and *b*_ii_ are regression coefficients for intercept, the coefficient of linear effects, interaction coefficient and coefficients of quadratic effect, respectively. *x*_i_ and *x*_j_ are coded value of the independent variables (i < j).

### The yield of GP-1 by *S. kanasenisi* ZX01 in bench fermentor

This part of the experiment was performed in a 5 L fermentor (GBCN-5C, Zhenjiang East Biotech Equipment and Technology Co., Ltd, China) with a working volume of 3 L. The fermentor was equipped with a temperature probe, pH sensor and dissolved oxygen (DO) sensor. The height and diameter of the 5 L fermentor were 0.35 and 0.2 m, respectively. The agitation system was a coupling of stirrer that consisted of two propellers on one axle and each propeller had four flat-blades. The agitation rate was controlled by electromagnetic impulse. The aeration system was an air inlet through a ring sparger with air-flow meter and filter. The fermentor and all its parts containing 3 litre medium was sterilized at 121 °C for 30 min. After sterilization, the fermentation medium was inoculated with 5% (v/v) seed medium. Diluted antifoaming agent was added when foam appeared in the fermentor during the fermentation process. The temperature and aeration rate were maintain at 28 °C and 3 L/min during the whole fermentation process, respectively. The agitation speed was controlled in the range of 150–300 rpm to maintain the DO concentrations over 20% saturation, which made sure that oxygen supply was enough for cell growth. Samples were acquired from fermentor at every 24 h interval for analysis of GP-1 production and dry cell weight (DCW).

## Results

### Effect of different media on the yield of GP-1

The effect of different media on GP-1 yield by strain ZX01 is presented in Table 2. The results indicated that The yield of GP-1 was the highest in millet medium (345.35 µg/L), which had significant differences with other media, followed by modified bean broth medium (280.47 µg/L), ISP2 (264.31 µg/L), bean broth medium (260.34 µg/L), ISP3 (244.60 µg/L), ISP5 (240.26 µg/L) and ISP4 (225.23 µg/L). On the other hand, GP-1 concentrations in Gause’s No.1 (190.74 µg/L), PDA (185.04 µg/L) and ISP1 (177.52 µg/L) were comparatively low and less than 200.00 µg/L. Among all the tested media, millet medium was chosen to be the optimal original medium for carbon and nitrogen sources selection experiments for the highest yield of GP-1.

**Table 2:** Effect of different media on the yield of GP-1 by strain ZX01

Media	The yield of GP-1 (µg/L)
Millet	345.35a
MBB	280.47b
ISP2	264.31b
BB	260.34b
ISP3	244.60bc
ISP5	240.26bc
ISP4	225.23c
Gause’s No. 1	190.74cd
PDA	185.04cd
ISP1	177.52d

Data in the table were average of three replicates, and different letters in each column indicated significant differences at the 5% level by Duncan’s multiple comparison

### Effect of different carbon and nitrogen sources on the yield of GP-1

Based on millet medium, the effect of different carbon and nitrogen sources on GP-1 yield by strain ZX01 is presented in Table 3. The results showed that *Streptomyces* sp. ZX01 had the maximum GP-1 yield with soluble starch (482.36 µg/L) as carbon source. Lactose (438.14 µg/L), sucrose (427.83 µg/L) and fructose (407.93 µg/L) were in the second echelon formation and had no significant difference with each other. In contrast, glycerol (211.28 µg/L) seemed to have negative influence on GP-1 yield.

**Table 3:** Effect of different carbon and nitrogen sources on the yield of GP-1 by strain ZX01

Carbon sources	The yield of GP-1 (µg/L)	Nitrogen sources	The yield of GP-1 (µg/L)
Soluble starch	482.36a	Yeast extract	534.83a
Lactose	438.14ab	Peptone	500.67ab
Sucrose	427.83bc	Soy peptone	471.42b
Fructose	407.93bc	Tryptone	456.71b
Glucose	366.56c	Beef extract	452.75b
Maltose	363.12c	Fish meal	378.58c
Glycerol	211.28d	Urea	352.20c

Data in the table were average of three replicates, and different letters in each column indicated significant differences at the 5% level by Duncan’s multiple comparison

As for the nitrogen sources, the maximum yield of GP-1 was obtained with yeast extract (534.83 µg/L) as nitrogen source. Peptone, soy peptone, tryptone and beef extract ranged from 500.67 to 452.75 µg/L. The yields of GP-1 with addition of fish meal (378.58 µg/L) and urea (352.20 µg/L) were lower than other nitrogen sources. Therefore, soluble starch and yeast extract were chosen as the optimal carbon and nitrogen source for the following experiments, respectively.

### Optimization of medium compositions

After confirmation of carbon and nitrogen source in fermentation medium, central composite design (CCD) was used to determine the optimal concentration of each composition of medium. The levels of the three independent variables, viz. soluble starch, yeast extract and inorganic salts are given in Table 1. A total of 20 experiments with different combinations of three independent variables were made according to CCD (Table 4). Each run was performed in triplicate and thus the experimental values of GP-1 yield given in Table 4 were averages of three sets of experiments, while the predicted values were obtained from quadratic polynomial equation mentioned below. The results were analyzed using Design Expert 8.0.5, and the following quadratic polynomial equation was found to explain the relationship between the independent variables and GP-1 yield:

5 $$\begin{aligned}Y &= 539.66 + 49.10x_{1} \\&\quad+ 24.54x_{2} + 12.58x_{3} - 2.83x_{1} x_{2} \\&\quad+ 6.93x_{1} x_{3} + 10.66x_{2} x_{3} \\&\quad- 22.24x_{1}^{2} - 13.90x_{2}^{2} - 14.94x_{3}^{2}\end{aligned}$$

where *Y* is the predicted response of GP-1 concentration, *x*_1_, *x*_2_ and *x*_3_ are coded values of soluble starch, yeast extract and inorganic salts, respectively.

**Table 4:** Experimental design by using CCD, experimental value and predicted value

Run	*x*_1_	*x*_2_	*x*_3_	Experimental value (µg/L)	Predicted value (µg/L)	Residual
1	−1	1	1	482.32	483.16	−0.84
2	0	0	0	536.84	539.66	−2.82
3	0	0	0	534.15	539.66	−5.51
4	−1	−1	1	397.65	407.10	−9.45
5	0	0	0	545.20	539.66	5.54
6	1	−1	−1	500.43	507.12	−6.69
7	−1	1	−1	438.56	450.54	−11.98
8	0	−1.682	0	465.94	459.06	6.88
9	0	0	0	532.35	539.66	−7.31
10	0	0	0	538.06	539.66	−1.60
11	−1	−1	−1	411.17	417.12	−5.95
12	0	1.682	0	445.41	541.61	3.80
13	0	0	−1.682	486.10	476.23	9.87
14	1	−1	1	529.24	524.82	4.42
15	0	0	0	549.52	539.66	9.86
16	1.682	0	0	556.87	559.33	−2.46
17	−1.682	0	0	407.31	394.15	13.16
18	1	1	−1	531.13	529.22	1.19
19	1	1	1	587.96	589.56	−1.60
20	0	0	1.682	519.37	518.55	0.82

In order to evaluate the significance and adequacy of the quadratic model, an analysis of variance (ANOVA) was conducted (Table 5). The ANOVA of the quadratic model indicates that the model is highly significant,due to the Fish’s *F* test (*F*_model_ = mean square regression/mean square residual = 68.92) with a very low *P* value [(*P*_model_ > *F*) < 00001]. The model’s goodness of fit can be checked by the determination coefficient (*R* ^2^) and the correlation coefficient (*R*). The *R* ^2^ value is always between 0 and 1. The closer the *R* ^2^ value is to 1, the better the correlation between the experimental and predicted values is (Wang et al. 2011). Here, the adj-*R* ^2^ (0.9699) demonstrates that about 97% of variability in the response is attributed to the independent variables and only about 3% of variability could not be explained by the model. The lack of fit measures the failure of the model to represent data in the experimental domain at point that are not included in the regression (Xu et al. 2008). The *F* value (3.06) of lack of fit is lower than the tabulated *F* value (*F*_0.01(9,5)_ = 10.15) and is not significant (*P* = 0.1227). The coefficient of variation (CV) is related with the precision and reliability, and the higher the value of CV the lower the reliability of experiment (Zhou et al. 2010). Here, a lower value of the CV (3.11%) indicates better precision and reliability of the experiment.

**Table 5:** The ANOVA of the quadratic model

Source	Sum of squares	Degree of freedom	Mean square	F value	P value (Prob > F)
Model	55,788.72	9	6198.75	68.92	<0.0001
Residual	899.41	10	89.94		
Lack of fit	677.75	5	135.55	3.06	0.1227
Pure error	221.66	5	44.33		
Total	56,688.13	19			

*R* ^2^ = 0.9841, adjusted *R* ^2^ = 0.9699, CV = 3.11%

The significant of each coefficient is determined by *t* value and *P* value, which are listed in Table 6. The student’s *t* test and *P* value also indicate the interaction strength between each independent variable. The larger the *t* value and smaller the *P* value is, the more significant the corresponding coefficient is. The results show that the linear coefficients of *X*_1_, *X*_2_ and quadratic coefficient of *X*_1_^2^ are more significant than the other factors, followed by *X*_3_, *X*_2_^2^ and *X*_3_^2^. These implied that the concentrations of soluble starch and yeast extract have strong influence on GP-1 yield. The interaction coefficients of *X*_1_ *X*_2_, *X*_1_ *X*_3_ and *X*_2_ *X*_3_ seem to be insignificant, which means that the interactions of any two variables have insignificant effect on GP-1 yield.

**Table 6:** Estimated regression coefficient and corresponding t value and P value

Factor	Coefficient estimate	Standard error	*t* value	*P* value
Intercept	539.66	3.87	–	–
*X*_1_	49.10	2.57	366.11	<0.0001
*X*_2_	24.54	2.57	91.44	<0.0001
*X*_3_	12.58	2.57	24.04	0.0006
*X*_1_ *X*_2_	−2.83	3.35	0.71	0.4184
*X*_1_ *X*_3_	6.93	3.35	4.27	0.0658
*X*_2_ *X*_3_	10.66	3.35	10.11	0.0098
*X*_1_^2^	−22.24	2.50	79.25	<0.0001
*X*_2_^2^	−13.90	2.50	30.96	0.0002
*X*_3_^2^	−14.94	2.50	35.76	0.0001

**Fig. 1:**
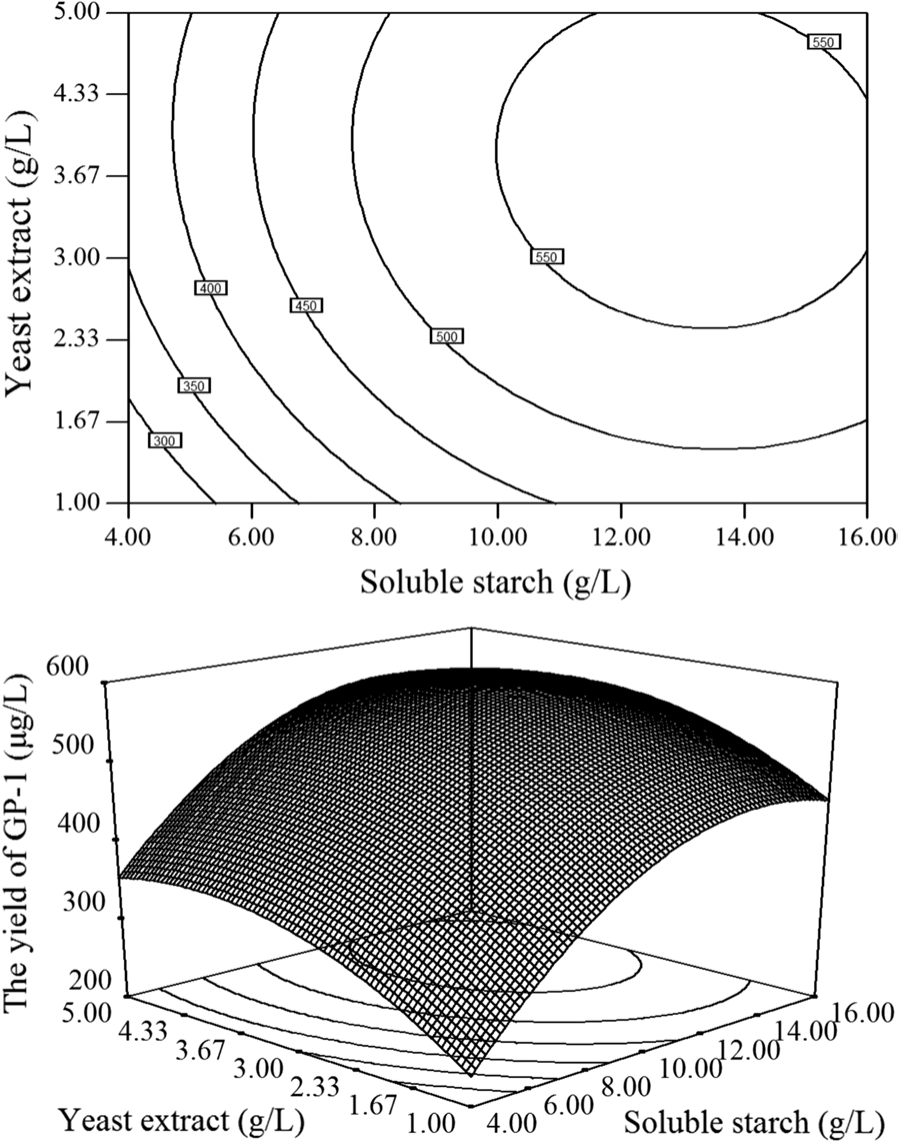
Contour plot and response surface plot of GP-1 yield: the combined effect of soluble starch and yeast extract on the yield of GP-1

**Fig. 2:**
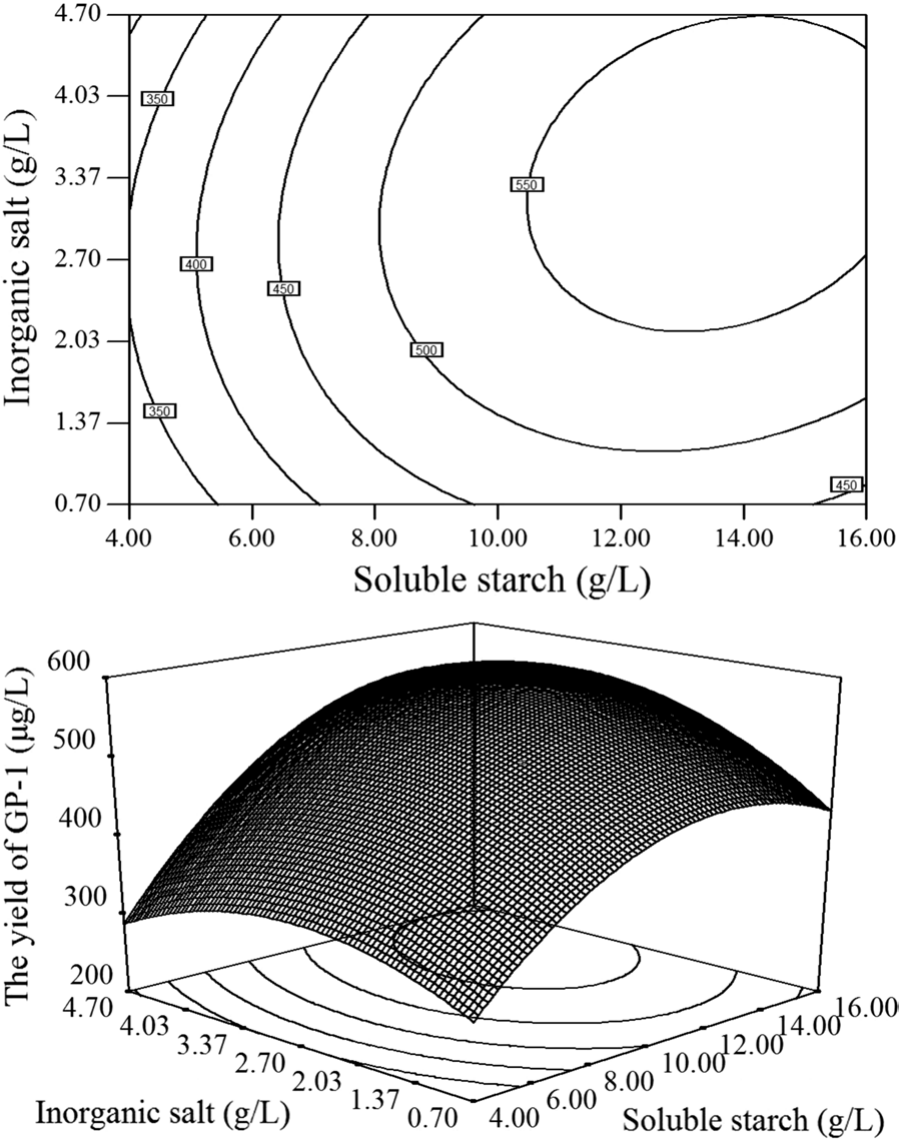
Contour plot and response surface plot of GP-1 yield: the combined effect of soluble starch and inorganic salts on the yield of GP-1

**Fig. 3:**
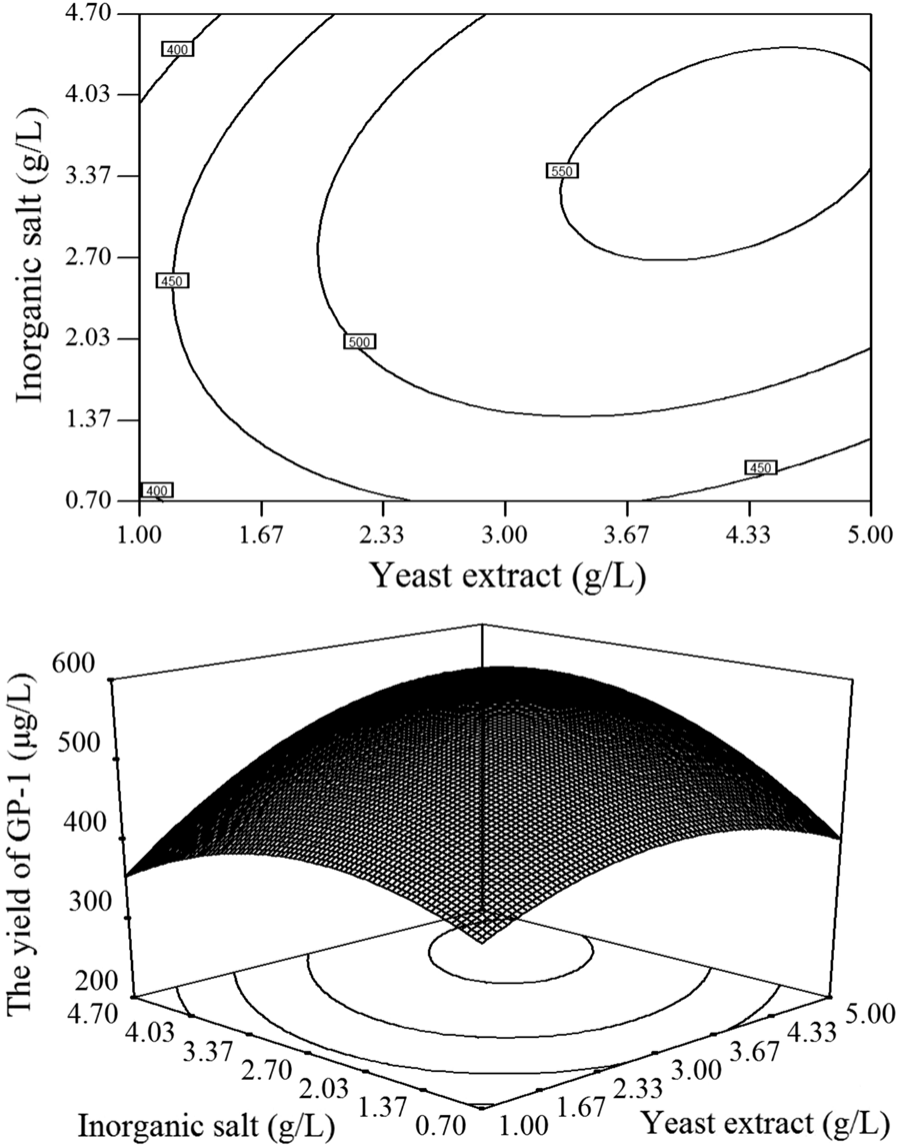
Contour plot and response surface plot of GP-1 yield: the combined effect of yeast extract and inorganic salts on the yield of GP-1

The 3D response surface plots and the 2D contour plots described by the regression model are drawn to expose the optimal values of the independent variables and interactive effects of each independent variable on the response. Both plots are presented in Figs. 1, 2 and 3. From the 3D response surface plots and the corresponding 2D contour plots, the optimal values of the independent variables and the maximum response could be predicted and the interaction between each independent variable could be understood. Each contour curve stands for a response value influenced by two test independent values with the rest maintained at its zero level. The maximum predicted value could be obtained in the smallest ellipse in 2D contour plots. The smallest ellipse also indicates that there is a perfect interaction between the independent values, optimal values of which are nearby (Xu et al. 2008; Yin et al. 2011).

As shown in Fig. 1, 2 and 3, the optimal values of medium compositions for obtaining the maximum yield of GP-1 lie in the following ranges: soluble starch 12–14 g/L, yeast extract 3.67–4.33 g/L and inorganic salts 3.37–4.03 g/L.

Through analysis of Design Expert Software, the optimal values of the independent variables in uncoded (actual) unit are: soluble starch 13.61 g/L, yeast extract 4.19 g/L and inorganic salts 3.82 g/L. Inorganic salts translated into NaCl and CaCO_3_ are 3.54 and 0.28 g/L. The model predicted that the maximum yield of GP-1 obtained by using the above optimal concentrations of medium compositions was 590.90 µg/L.

A verification experiment using the optimized medium was carried out. The maximum yield of GP-1 was found to be 601.33 µg/L, which is in close agreement with the model prediction (590.90 µg/L). This indicated that the model was suitable and accurate for enhancing the yield of GP-1 by strain ZX01. The yield of GP-1 using the optimized medium (soluble starch 13.61 g/L, yeast extract 4.19 g/L, NaCl 3.54 g/L, CaCO_3_ 0.28 g/L, millet 10 g/L) was 601.33 µg/L, while the yield of GP-1 using the original medium (glucose 10.00 g/L, peptone 3.00 g/L, NaCl 2.50 g/L, CaCO_3_ 0.20 g/L, millet 10 g/L) was 345.35 µg/L. After optimization, the yield of GP-1 was improved by 74.12%. Moreover, a anti-TMV activity assay was also conducted, and the anti-TMV activity through optimization was 91.10%, in contrast with 74.88% using original millet medium.

### Fermentation in 5 L bench fermentor

**Fig. 4:**
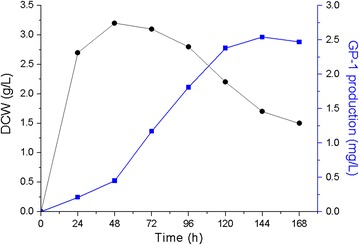
Time course of GP-1 production and DCW by *S. kanasenisi* ZX01 using optimized medium in 5 L fermentor (squares is GP-1 production, circles is DCW)

Based on the medium obtained from shake flask optimization experiments, a scale-up fermentation was conducted in a 5 L bench fermentor. A time course of GP-1 production and DCW was presented in Fig. 4. DCW increased sharply to a maximum value (3.2 g/L) at 48 h, and then decreased to 1.5 g/L at the end of the fermentation process. The highest yield of GP-1 was achieved at 120 h and was 2.54 mg/L, which is about 4.23 times higher than that using optimized medium in shake flask (601.33 µg/L), and 7.36 times higher than that using original medium (345.35 µg/L).

## Discussion

GP-1 is a novel glycoprotein produced by *S. kanasenisi* ZX01 with significant activity against TMV (Zhang et al. 2015). However, the extremely low yield of GP-1 and long fermentation period of strain ZX01 have restricted its further researches and market applications. For this reason, this paper was aimed at improving the yield of GP-1, which laid the foundation for developing GP-1 to be a novel anti-plant virus agent. Any microbial fermentation process can be affected by medium compositions and process parameters, and therefore statistical experimental design methods were used efficiently to optimize fermentation medium of strain ZX01. Among 10 different media, which have been reported earlier for the growth of *Streptomyces*, millet medium was selected to be the original fermentation medium. Based on the millet medium, one-factor-at-a-time optimization was used to determine carbon source and nitrogen source. The comparison of several commonly used carbon sources indicated that soluble starch was the optimal carbon source. It is well known that carbohydrates are energy source of the organism and play a key role in the metabolite biosynthesis, thus the yield of GP-1 was improved by 39.67% compared with the original medium. We also found that the polysaccharides as carbon sources were better than monosaccharides and disaccharides for the strain ZX01 to produce a higher yield of GP-1. The reason may be that soluble starch is hydrolyzed to glucose slowly in liquid medium and the rate is very slow compared with that of glucose uptake, leading to alleviation of catabolite repression on growth caused by glucose (Chen et al. 2010). Similarly, yeast extract was screened out to be the best nitrogen source. Yeast extract is an ideal organic nitrogen source in the fermentation industry, since it is inexpensive and could be more easily absorbed by microorganisms (Hernández-Cortés et al. 2016). The optimal concentrations of carbon source, nitrogen source and inorganic salts were further optimized by RSM based on CCD. RSM was proved to be a powerful tool for optimizing GP-1 yield by strain ZX01. The RSM model equation demonstrated that soluble starch, yeast extract and inorganic salts were positively significant factors to GP-1 production. From the equation, interaction between two factors was also found. Both soluble starch and yeast extract interacted positively with inorganic salts, whereas there was negative interaction between soluble starch and yeast extract. The final optimized fermentation medium was shown as follows: soluble starch 13.61 g/L, yeast extract 4.19 g/L, NaCl 3.54 g/L, CaCO_3_ 0.28 g/L, millet 10 g/L. Theoretically, the predicted value of GP-1 production could reach 590.90 µg/L using this medium. In practice, the maximum yield of GP-1 was found to be 601.33 µg/L in verification test, which also proved that the model was able to predict GP-1 yield accurately. Compared with original fermentation medium, the yield of GP-1 by strain ZX01 has increased by 74.12% after optimization.

A scale-up fermentation of *S. kanasenisi* ZX01 was further carried out in 5 L bench fermentor. The yield of GP-1 was improved to 2.54 mg/L when the DO was sufficient, which was much higher than that in shake flask. There is a big difference between fermentor and shake flask in agitation and aeration form, therefore, the reason why the yield of GP-1 in 5 L fermentor was much higher than that in shake flask might be the oxygen transfer rate (OTR). OTR is an important parameter rely on the operation of a reaction and agitation in fermentor (Bandaiphet and Prasertsan 2006; Mantzouridou et al. 2002). This result provides a useful idea for the improvement of GP-1 production in 5 L bench fermentor through optimizing aeration and agitation.

Our medium optimization study provides first-hand data and informations that are fundamental and useful for the development of strain ZX01 fermentation process and improvement of GP-1 production on a large scale. Moreover, due to the rapid development of modern bio-engineering technology, these informations make it possible for the strain ZX01 to become an efficient engineering strain combined with genetic engineering methods, which provides us more glycoproteins with activity against plant virus, and even various natural products for pesticides discovery.

## Abbreviations

RSM: response surface methodology
CCD: central composite design
TMV: tabacco mosaic virus
ANOVA: analysis of variance
DO: dissolved oxygen
DCW: dry cell weight
OTR: the oxygen transfer rate
HPLC: high performance liquid chromatography
